# Oxyresveratrol in Breast Cancer Cells: Synergistic Effect with Chemotherapeutics Doxorubicin or Melphalan on Proliferation, Cell Cycle Arrest, and Cell Death

**DOI:** 10.3390/pharmaceutics16070873

**Published:** 2024-06-29

**Authors:** Carlos Luan Alves Passos, Christian Ferreira, Aline Gabrielle Alves de Carvalho, Jerson Lima Silva, Rafael Garrett, Eliane Fialho

**Affiliations:** 1 Nutrition Institute Josué de Castro, Federal University of Rio de Janeiro, Rio de Janeiro 21941-902, RJ, Brazil; luh.passos@live.com (C.L.A.P.); christianferreira_83@hotmail.com (C.F.); 2 Chemistry Institute, Federal University of Rio de Janeiro, Rio de Janeiro 21941-909, RJ, Brazil; alinegac@gmail.com (A.G.A.d.C.); rafael_garrett@iq.ufrj.br (R.G.); 3 Medical Biochemistry Institute Leopoldo De Meis, Federal University of Rio de Janeiro, Rio de Janeiro 21941-902, RJ, Brazil; jerson@bioqmed.ufrj.br

**Keywords:** breast cancer, oxyresveratrol, doxorubicin, melphalan

## Abstract

Breast cancer is the second most common type of cancer in the world. Polyphenols can act at all stages of carcinogenesis and oxyresveratrol (OXY) promising anticancer properties, mainly associated with chemotherapy drugs. The aim of this study was to investigate the effect of OXY with doxorubicin (DOX) or melphalan (MEL), either isolated or associated, in MCF-7 and MDA-MB-231 breast cancer cells. Our results showed that OXY, DOX, and MEL presented cytotoxicity, in addition to altering cell morphology. The synergistic association of OXY + DOX and OXY + MEL reduced the cell viability in a dose-dependent manner. The OXY, DOX, or MEL and associations were able to alter the ROS production, ∆Ψm, and cell cycle; DOX and OXY + DOX led the cells to necrosis. Furthermore, OXY and OXY + MEL were able to lead the cells to apoptosis and upregulate caspases-3, -7, -8, and -9 in both cells. LC-HRMS showed that 7-deoxidoxorubicinone and doxorubicinol, responsible for the cardiotoxic effect, were not identified in cells treated with the OXY + DOX association. In summary, our results demonstrate for the first time the synergistic effect of OXY with chemotherapeutic agents in breast cancer cells, offering a new strategy for future animal studies.

## 1. Introduction

Cancer is a growing health problem and is one of the leading causes of death. According to WHO, in 2020, there were 2.3 million women diagnosed with breast cancer and 685.000 deaths globally; therefore, breast cancer is the type of cancer that kills the most women in the world, particularly in low- and middle-income countries [1]. Current cancer treatments include cytotoxic chemotherapy; however, some limitations are observed, such as high toxicity and resistance to chemotherapy [2].

Phytochemicals from food sources have proven to be a good alternative in the treatment of breast cancer, displaying many anticarcinogenic properties, including inhibitory effects on cell proliferation, angiogenesis, and metastasis [3]. Human consumption associated with the absence of systemic toxicity and unfavorable side effects make these bioactive compounds promising candidates for the treatment of breast cancer. Furthermore, these phytochemicals combined with chemotherapy drugs have proven to be a favorable alternative in cancer treatment. The benefits from the combination include an increase in bioavailability and lower concentrations, mitigating the toxic effects of anticancer drugs [4,5].

A combination of phytochemicals and anticancer drugs could increase the effectiveness of chemotherapy by overcoming drug resistance and reducing toxicity and side effects [6]. The polyphenol resveratrol (RESV) present in grapes demonstrated a synergistic effect with doxorubicin (DOX); the combination reduced the dose of DOX by 2.5 times compared to the IC_50_ concentration alone, inhibiting survival and invasion, in addition to inducing apoptosis in MCF-7 and MDA-MB-231 breast cancer cells [7]. Furthermore, RESV potentiated the cytotoxic effects of melphalan (MEL) in MCF-7 breast cancer cells, inducing death by apoptosis and cell cycle arrest in the S phase [8].

Oxyresveratrol (OXY; trans-2,3’,4,5’-tetrahydroxystilbene) is a naturally occurring phenolic compound found in several species, among them *Morus nigra* L. (blackberry). OXY has a simple chemical structure, containing a trans-1,2-diphenylethylene nucleus with two -OH groups on each of the aromatic rings, and it has diverse therapeutic potential, with as antioxidant, anti-inflammatory, and anticancer effects [9].

Evidence indicates the effect of OXY on several molecular targets in different cancer cells. In MCF-7 cells, OXY induces apoptosis and cell cycle arrest in the G1 and S phases, in addition to regular genes included in DNA components, highlighting that this polyphenol can be used to overcome drug resistance and increase the effectiveness of chemotherapy drugs [9,10,11]. However, no studies have been found with this phytochemical in combination with chemotherapeutic agents. Therefore, studies are needed to demonstrate the potential antitumor properties of OXY which are either associated or not with anticancer drugs such as DOX or MEL in breast cancer cells.

In the present study, we investigated the cytotoxic effect of OXY, evaluating possible mechanisms of action on MCF-7 and MDA-MB-231 breast cancer cells, in addition to demonstrating the synergistic association of this phytochemical with the chemotherapeutic agents DOX and MEL for the first time, thus providing promising outcomes for the utilization of OXY, either isolated or associated with anticancer drugs, as a novel template for future in vivo and clinical studies of breast cancer therapeutics.

## 2. Materials and Methods

### 2.1. Reagents

Oxyresveratrol (*trans*-2,3′,4,5′-tetrahydroxystilbene, OXY), melphalan (MEL), DMSO (dimethyl sulfoxide), 2′,7′-dichlorofluorescin diacetate (DCFDA), and JC-1 solution were purchased from Sigma-Aldrich (St. Louis, MO, EUA). The annexin-V apoptosis detection kit was purchased from Molecular Probes (Carlsbad, CA, USA). Primary antibody anti-caspase-3, anti-caspase-7, anti-caspase-8, and anti-caspase-9 were purchased from Santa Cruz (Dallas, TX, USA), and the secondary antibody Alexa 488 was purchased from TermoFisher (Waltham, MA, USA). Doxorubicin chloridrate (DOX) was donated by Dr. Raquel Maia (Instituto Nacional de Câncer José Alencar Gomes da Silva—INCA, RJ, Brazil).

### 2.2. Cell Cultures

The following human breast epithelial cell lines, obtained from the American Type Culture Collection (ATCC; Manassas, VA, USA), were used in this study: MDA-MB-231, as estrogen receptor-negative cell line derived from a metastatic carcinoma; MCF-7, an estrogen receptor-positive cell line derived from an in situ carcinoma; and MCF-10A, a non-tumor breast cell line. MDA-MB-231 and MCF-7 cells were maintained in DMEM medium supplemented with 10% fetal bovine serum. MCF-10A was maintained in DMEM/F12 medium supplemented with 10% fetal bovine serum, insulin 0.01 mg/mL, EGF 0.02 mg/mL, and hydrocortisone 0.05 mg/mL. Both media contained 100 units/mL of penicillin and 100 μg/mL of streptomycin. The cell cultures were kept at 37 °C in a humidified atmosphere of 5% CO_2_ in air.

### 2.3. Cell Viability Assay

The cell viability assay was performed using MTT. Briefly, cell cultures were treated with different concentrations of OXY, DOX, and MEL for 24 h. After treatment, cells were washed with phosphate-buffered saline (PBS) and then incubated for 3 h in 0.5 mL of MTT solution (0.5 mg/mL of PBS) at 37 °C in 5% CO_2_ in an incubator. The medium was removed, and DMSO 0.5 mL was added to each well to dissolve the resulting formazan crystals. The absorbance was measured at a wavelength of 595 nm [12]. The results are expressed in as a percentage of viable cells compared to the untreated control.

### 2.4. Selectivity Index Calculation (SI)

The selectivity index was calculated as the ratio of non-tumor MCF-10A IC_50_ cells to MCF-7 IC_50_ or MDA-MB-231 IC_50_ cancer cells, as described by Passos et al. (2015) [13] and modified.

### 2.5. Isobologram Construction

The fractional inhibitory concentration index (FIC) of OXY was calculated using the formula IC_50_ of OXY in association with the IC_50_ of OXY alone (IC_50_, 50% inhibitory concentration) in MCF-7 or MDA-MB-231 cells. The same formula was applied to DOX and MEL. The mean FIC was calculated for each combination and then compared to the reference values and reported as very strong synergism (<0.1), strong synergism (0.1–0.3), synergism (0.3–0.7), moderate synergism (0.7–0.85), slight synergism (0.85–0.90), nearly additive (0.90–1.10), slight antagonism (1.10–1.20), moderate antagonism (1.20–1.45), antagonism (1.45–3.3), strong antagonism (3.3–10), or very strong antagonism (>10). The synergistic effects between OXY + DOX and OXY + MEL were analyzed using FIC values to plot the isobologram, according to the method of Chou (2006) [14]. Antagonistic combinations were defined as points above the line of additivity, and synergistic combinations were defined as points below the line.

### 2.6. Test of Colony Formation (CFU)

The test of colony formation was performed using crystal violet according to the method described by Guimarães et al. (2017), which was modified [15]. The MCF-7 or MDA-MB-231 cells were adjusted at a density of 10^3^ cells/per well in a 24-well plate for 24 h. The cells were treated with OXY, DOX, MEL, and associations at their respective ICs_50_ concentrations for 24 h, with medium replacement every 3 days. After 18 days, colonies were fixed in methanol for 10 min and then stained with 5% crystal violet for 30 min at room temperature. For colonic analyses, they were washed five times with PBS for 5 min, and 50 cells were counted using an inverted microscope.

### 2.7. Detection of Reactive Oxygen Species

The detection of reactive oxygen species (ROS) was performed using MCF-7 or MDA-MB-231 cells adhered to 6-well opaque culture plates and treated with OXY, DOX, MEL, and associations at their respective ICs_50_ concentrations for 3 h. The cells were then stained with 50 μM DCFDA, and ROS was measured immediately using 500/526 nm excitation/emission wavelengths using a BD FACSCalibur™ flow cytometer (Becton Dickinson, Franklin Lakes, NJ, USA), then analyzed by the BD CellQuest™ Pro software v.3.3 (Becton Dickinson) [12].

### 2.8. Measurement of the Mitochondrial Membrane Potential

The MCF-7 or MDA-MB-231 cells were treated or not with IC_50_ of OXY, DOX, MEL, and associations for 24 h, and then incubated with a JC-1 solution (5 μg/ml) for 20 min at 37 °C according to the manufacturer’s instructions. The mitochondrial membrane potential (∆Ψm) was measured in 6-well plates using 490/530 nm excitation/emission (JC-1 monomers) and 525/590 nm excitation/emission (J-aggregates) using a BD FACSCalibur™ flow cytometer (Becton Dickinson, Franklin Lakes, NJ, USA), then analyzed using BD CellQuest™ Pro software v.3.3 (Becton Dickinson), as described by Costa et al. (2022) [16].

### 2.9. Cell Cycle Analysis

The MCF-7 or MDA-MB-231 cells were treated with OXY, DOX, MEL, and associations at their respective ICs_50_ concentrations for 24 h. After treatment, the cells (5 × 10^5^) were washed with PBS and fixed in 70% (*v/v*) ice-cold methanol/PBS for at least 1 h at 4 °C. The fixed cells were washed once with PBS and then incubated in PBS supplemented with 10 μg/mL propidium iodide (PI) and 20 μg/mL RNAse at 37 °C for 30 min at room temperature in the dark. For each sample, 10,000 events were analyzed using a FACScan (Becton and Dickson) and the CellQuest software v.3.3.

### 2.10. Annexin V Binding Assay

Double staining for annexin V-fluorescein isothiocyanate (FITC) and PI was performed with the annexin-V apoptosis detection kit. MCF-7 or MDA-MB-231 cells were treated or not with different concentrations of OXY, DOX, MEL, and associations for 24 h. They were then washed twice in cold annexin V-buffer and centrifuged at 2000 rpm for 5 min Pellets were resuspended in 20 μL of annexin V-FITC, and after 15 min of incubation in the dark, 480 μL of annexin V-buffer was added according to the manufacturer’s instructions. Annexin V-FITC labeling was recorded on a BD FACScalibur (Becton and Dickson) and analyzed using CellQuest software v.3.3.

### 2.11. Immunocytochemistry

The cells were treated with OXY, DOX, MEL, and associations at their respective ICs_50_ concentrations for 24 h. Next, the cells were fixed with 4% paraformaldehyde, 0.3% BSA and Triton X-100 0.5% for 20 min and permeabilized with Triton X-100 (0.5% in PBS) for 25 min. The MCF-7 and MDA-MB231 cells were then washed in PBS three times, and the primary antibody for apoptosis (anti-caspase-3, anti-caspase-7, anti-caspase-8, or anti-caspase-9) was added and incubated in the dark for approximately 1 h. The cells were then washed in PBS three times, and the fluorescence secondary antibody Alexa 488 was added and incubated in the dark for approximately 1 h. The cells were washed and collected by flow cytometry BD FACSCalibur (Becton, Dickinson), then analyzed using the CellQuest software v.3.3 [12].

### 2.12. Samples and Analyte Extraction

A methanolic extraction was performed by adding 1 × 10^7^ MCF-7 and MDA-MB-231 cells treated with OXY, DOX, MEL, and associations at their respective ICs_50_ concentrations for 24 h. Sample extracts were dried for 40 min in a speedvac (RVC 2–25 CDPlus; Christ, Germany), resuspended in 120 μL of methanol:H_2_O solution (80:20, *v/v*) containing *p*-fluoro-phenylalanine at 2 μg/mL^−1^, and centrifuged (MySpin 12; Thermo Scientific, Waltham, MA, USA) at 5080 g for 10 min, and then 100 μL of the supernatant was collected for analysis. Control cells without any type of treatment and cells with the addition of DMSO (vehicle control) were also analyzed.

### 2.13. Standards and Their Metabolites

OXY, DOX, and MEL were prepared at a concentration of 2 μg/mL^−1^ in methanol:H_2_O (80:20, *v/v*) and analyzed in the same conditions as the cell samples to help with their identification. A retention time error of 0.01 min, a mass error of 5 ppm, and their fragmentation spectra were considered for identification. In addition, 16 metabolite candidates from these standards were investigated in cell samples based on their theoretical *m/z* ions, as listed in Supplementary Table S1.

### 2.14. Chromatographic Method

Standards and samples were injected into a liquid chromatographer (Dionex Ultimate 3000; Thermo Fisher Scientific, Bremen, Germany) coupled to a high-resolution mass spectrometer (Q-Exactive Plus Orbitrap; Thermo Fisher Scientific, Bremen, Germany) with an electrospray ionization source (ESI). A Waters Acquity UPLC BEH C18 (1.7 μm, 2.1 × 100 mm) column was used for metabolite separation at 40 °C. The mobile phases consisted of (A) water and (B) methanol, both with 0.1% of formic acid and flow rates of 350 μL min^−1^. The elution gradient was 0 to 1 min, 5% B; 1 to 9 min, 5–60% B; 9–13 min, 60–98% B; 13 to 17 min, 98% B (column washing); and 17.1 to 20.0 min, 5% B (column equilibration to the initial conditions). The overall run time was 20 min, and the injection volume was 4 μL. The LC effluent was pumped to the mass spectrometer, and the ESI ion source was operated in positive-ion mode with a spray voltage of 3.9 kV, capillary temperature of 320 °C, sheath gas of 45 arbitrary units (a.u.), auxiliary gas of 15 a.u. (both nitrogen), probe heater temperature of 400 °C, and S-Lens RF Level 60.00. Data were acquired he range of *m/z* 100–1000 at a resolution of 35,000 FWHM (full width at half maximum). The automatic gain control (AGC) target was 1.0e6, with a maximum injection time (IT) of 180 ms.

### 2.15. In Silico Analyses

In silico theoretical analyses were carried out as proposed by Garcia et al. (2023) [17]. The chemical structures of OXY, DOX, MEL, and the metabolites doxorubicionol and 7-deoxydoxorubicinone were obtained from the PubChem database (https://pubchem.ncbi.nlm.nih.gov/, accessed on 19 May 2024) in SDF 2D format. In silico ADMET analyses were evaluated using the PreADMET web server (https://preadmet.qsarhub.com/, accessed on 19 May 2024), and different descriptors were evaluated, such as solubility, octanol/water coefficient partition (AlogP98), plasma protein binding, human intestinal absorption, intestinal and kidney cell permeability, skin permeability, blood–brain barrier penetration, CYP inhibition, and substrate and oral bioavailability (rule of five). The toxicological analysis included mutagenicity (Ames test), carcinogenicity, and inhibition of human Ether-a-go-go-related genes (hERG).

### 2.16. Statistical Analysis

Data were analyzed using Student’s t-test when comparing two groups or one-way ANOVA for more than two groups using the software GraphPad Prism 6.0. *p* values less than 0.05 were considered statistically significant.

## 3. Results

### 3.1. Oxyresveratrol and Chemotherapeutic Agents Doxorubicin and Melphalan against Non-Tumor Breast Cells and Breast Cancer Cells

**Figure 1:**
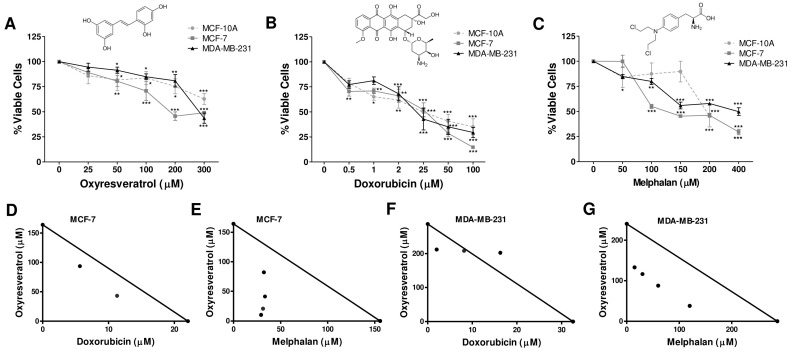
Cytotoxicity effects of isolated oxyresveratrol, doxorubicin, and melphalan and their associations on breast cancer cells. The non-tumor breast cell line MCF-10A and breast cancer MCF-7 and MDA-MB-231 cells were treated with different concentrations of oxyresveratrol (OXY) (**A**), doxorubicin (DOX) (**B**), and melphalan (MEL) (**C**) for 24 h, and then cell viability was assessed using an MTT assay. In the figure set: the chemical structures of OXY, DOX, and MEL. For the isobolographic analysis, MCF-7 cells were grown in the presence or absence of the indicated concentrations of OXY + DOX (**D**) and OXY + MEL (**E**). Similarly, MDA-MB-231 cells were grown in the presence or absence of the indicated concentrations of OXY + DOX (**F**) and OXY + MEL (**G**). Cytotoxicity for cells was determined by the MTT assay after 24 h of treatment. The straight line is the line of additivity and represents all of the additive theoretical combinations that should inhibit survival by 50%. The points below the additivity line represent synergistic combinations. The results represent the means ± SEM from three experiments performed in triplicate. * *p* < 0.05, ** *p* < 0.001, *** *p* < 0.0001, in relation to the control.

Initially, we performed the cytotoxicity assay to assess the effect OXY on breast cancer cell lines MCF-7 and MDA-MB-231 and non-tumor breast cell line MCF-10A. After treatment with different concentrations of compounds for 24 h, the MTT assay was performed (Figure 1). We observed that OXY reduced the cell viability of the MCF-7 and MDA-MB-231 cells in a dose-dependent manner. The cytotoxicity in MCF-7 and MDA-MB-231 cells started at the concentration of 50 μM, with significant reductions of 24.63 and 8.58% (*p* < 0.05), respectively (Figure 1A).

Furthermore, we used two classical chemotherapeutic agents, DOX (anthracycline antibiotic) and MEL (nitrogen mustard alkylating agent). The DOX significantly reduced the viability of MCF-7 and MDA-MB-231 by 29.55 and 31.98% (*p* < 0.001) at concentrations of 0.5 and 2 μM, respectively (Figure 1B). MEL, with concentrations of 100 and 50 μM, significantly reduced the viability of MCF-7 and MDA-MB-231 cells by 45.00 and 15.24% (*p* < 0.05), respectively (Figure 1C), which demonstrates a more pronounced cytotoxic effect on MCF-7 cells. In human non-tumor mammary MCF-10A cells, the OXY, DOX, and MEL, at 100 µM, reduced the viability cells by 16.41, 64.89, and 12.57%, respectively (Figure 1A–C).

The IC_50_ values of OXY, DOX, and MEL were determined, with values of >300, 26.54, and 195.97 µM for MCF-10ª; 164.10, 22.60, and 155.70 µM for MCF-7; and 287.08, 32.55, and 240.26 µM for MDA-MB-231 cells, respectively (Table 1). The selectivity index (SI) was also calculated from the IC_50_ obtained from the MCF-10A cells and that from the MCF-7 and MDA-MB-231 cells. The SI values for OXY were >1.83 and >1.05, while for DOX, they were 1.17 and 1.26, and for MEL, they were 0.82 and 0.82, respectively (Table 1).

### 3.2. Isobolographic Analysis of the Oxyresveratrol + Doxorubicin and Oxyresveratrol + Melphalan Associations in Breast Cancer Cell Lines

It has been commonly assumed that the combination of chemotherapy for breast cancer with true synergistic potential would result in an improved response rate and better symptom palliation and survival compared to single-agent chemotherapy [18]. Therefore, we assessed whether OXY could synergize with DOX and MEL using the fractional inhibitory concentration index and the isobologram method. Thus, the IC_50_ values for both drugs were determined (Table 2). The calculated IC_50_ value for OXY was plotted on the *y* axis, and the IC_50_ value for DOX was plotted on the *x* axis (Figure 1D,F). For MEL, the IC_50_ value was plotted on the *x* axis (Figure 1E,G). The two points were connected to form the line of additivity. The points on this line were classified as additive; points above the line indicated antagonistic effects, and points below the line indicated synergistic effects. The combination of 93.61 µM OXY + 5.65 µM DOX in MCF-7 and 211.09 µM OXY + 2.034 µM DOX in MDA-MB-231 showed a moderate synergism effect, with FIC values of 0.82 and 0.80, respectively. Likewise, the combination of 10.25 µM OXY + 29.25 µM MEL in MCF-7 cells showed strong synergism, and 132.93 µM OXY + 15.01 µM MEL in MDA-MB-231 cells presented synergism, with FIC values of 0.25 and 0.52, respectively (Table 2).

Our results demonstrated that 93.61 µM OXY + 5.65 µM DOX in MCF-7 and 211.09 µM OXY + 2.034 µM DOX in MDA-MB-231 cells were able to decrease the concentrations of DOX by 4.0 and 16.0 times, respectively. Meanwhile, the associations of 10.25 µM OXY + 29.25 µM MEL in MCF-7 cells with 132.93 µM OXY + 15.01 µM MEL in MDA-MB-231 cells were able to decrease the concentrations of this chemotherapeutic agent by 5.0 and 16.0 times, respectively, without them losing their activity when compared separately (Table 2).

### 3.3. Formation of MCF-7 and MDA-MB-231 Colonies after Treatment with Oxyresveratrol, Doxorubicin, Melphalan, and Associations

**Figure 2:**
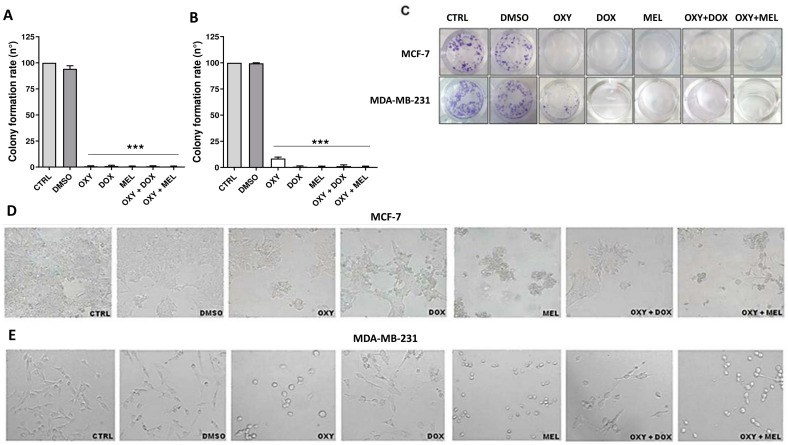
Formation of MCF-7 and MDA-MB-231 colonies and morphological analysis after treatment by oxyresveratrol, doxorubicin, melphalan, and associations. After treatment with IC_50_ concentrations of oxyresveratrol (OXY), doxorubicin (DOX), melphalan (MEL), OXY + DOX, and OXY + MEL or 1% DMSO for 24 h, the numbers of MCF-7 (**A**) and MDA-MB-231 **(B**) colonies were determined after 15 days of culture. MCF-7 and MDA-MB-231 (**C**) cells were plated and treated with OXY, DOX, MEL, OXY + DOX, and OXY + MEL for 24 h, with the medium replaced every 3 days. Cell colonies were stained with crystal violet. Data are presented as the mean ± SEM of three independent experiments. *** *p* < 0.0001 in relation to control. For the morphological analysis, the MCF-7 (**D**) and MDA-MB-231 cells (**E**) were treated with OXY, DOX, MEL, OXY + DOX, and OXY + MEL combinations for 24 h. Images are representative of three independent experiments with 100× magnification.

To further examine the cytotoxic effects of OXY, DOX, MEL, and associations over a prolonged period, clonogenic assays were performed on both cell lines for 18 days after treatment with IC_50_, respectively. According to the literature, cell groups with more than 50 cells were considered as colonies [15]. Our data showed that the clonogenic ability of MCF-7 and MDA-MB-231 cells was inhibited in the presence of IC_50_ of OXY, DOX, MEL, and associations of OXY + DOX and OXY + MEL (Figure 2A–C).

### 3.4. Morphological Analysis of Breast Cancer Cells after Treatment with Oxyresveratrol, Doxorubicin, Melphalan, and Associations

Light microscopy was used to evaluate the morphological effects of OXY, DOX, MEL, and their associations on MCF-7 and MDA-MB-231 cells. MCF-7 and MDA-MB-231 cells were incubated with the compounds for 24 h at the determined IC_50_ values. The treatment of OXY in MCF-7 cells showed no significant morphological changes, but reduced the cell density. Regarding the treatment with DOX, MEL, and OXY + DOX and OXY + MEL combinations, it was possible to observe changes in cell morphology, with a reduction in cell size in relation to the control (Figure 2D). In addition, there was fragmentation and formation of cell membrane extensions. OXY treatment on MDA-MB-231 cells resulted in morphological changes that included rounding and retraction of cells, with reduced cell size compared to the control. The same pattern of change in cell morphology was observed after treatment with DOX and the associations OXY + DOX and OXY + MEL. With all treatments, a decrease in cell density was observed (Figure 2E).

### 3.5. Effects of Oxyresveratrol, Doxorubicin, Melphalan, and Associations on Production of Reactive Oxygen Species and Alteration of Mitochondrial Membrane Potential in Breast Cancer Cell Lines

**Figure 3:**
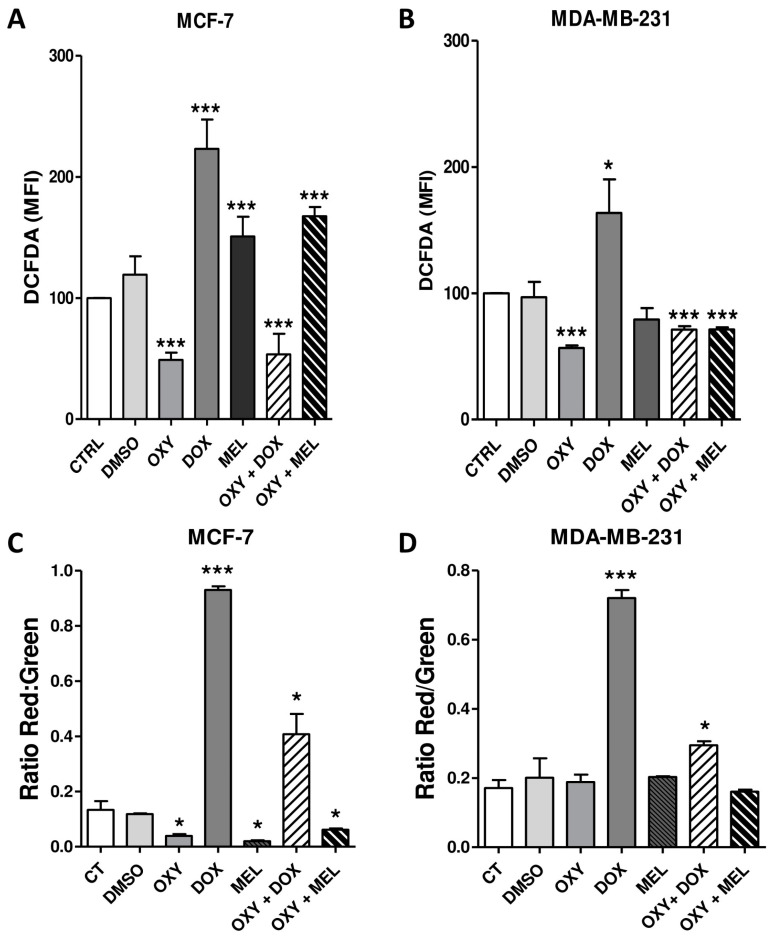
The effects of oxyresveratrol (OXY), doxorubicin (DOX), melphalan (MEL), and associations on production of reactive oxygen species (ROS) and mitochondrial membrane potential (ΔΨm) in MCF-7 and MDA-MB-231 cells. Detection of ROS was performed using MCF-7 (**A**) and MDA-MB-231 (**B**) cells adhered to 24-well culture plates and treated with OXY, DOX, MEL, and associations or 1% DMSO at their respective IC_50_ concentrations for 3 h. The cells were then stained with 50 μM 2′,7′-dichlorofluorescin diacetate (DCFDA, Sigma-Aldrich), and ROS was measured immediately using flow cytometry. Detection of ΔΨm was performed using MCF-7 (**C**) and MDA-MB-231 (**D**) cells adhered to 24-well culture plates and treated with OXY, DOX, MEL, and associations or 1% DMSO at their respective IC_50_ concentrations for 3 h, the cells were evaluated after JC-1 staining by flow cytometry analysis, and the results were expressed as red/green fluorescence ratios. The data represent the mean ± SEM of three independent experiments. * *p* < 0.05, *** *p* < 0.0001, in relation to control.

We found that DOX increased the ROS production by 1.13- and 1.89-fold in MCF-7 and MDA-MB-231 cells, respectively. However, the OXY + DOX association in both cells reduced ROS levels by 1.87- and 1.41-fold (Figure 3A,B). Similarly, treatment with 164.10 µM OXY in MCF-7 and 287.08 µM in MDA-MB-231 cells reduced ROS levels by 0.50- and 1.71-fold (Figure 3A,B). However, a difference in ROS production was observed in MCF-7 and MDA-MB-231 cells after treatment with MEL and OXY + MEL. We found that 155.70 µM MEL and 10.25 µM OXY + 29.25 µM MEL increased ROS production by 1.51- and 1.78-fold in MCF-7 cells (Figure 3A). However, in MDA-MB-231 cells, the association of 132.93 µM OXY + 15.01 µM MEL reduced ROS levels by 1.40-fold (Figure 3B).

Mitochondrial membrane potential (ΔΨm) directly or secondarily influences the control of redox and pH microenvironments, as well as proliferation and cell death [19]. Our results showed increases of 6.96- and 3.05-fold and 4.19- and 1.72-fold in the ΔΨm in MCF-7 and MDA-MB-231 cells treated with 22.60 µM of DOX and 93.61 µM OXY + 5.65 µM DOX, respectively (Figure 3C,D). On the other hand, the treatment with 164.10 µM OXY, 155.70 µM MEL, and 10.25 µM OXY + 29.25 µM MEL in MCF-7 cells reduced the ΔΨm by 3.42-, 6.54-, and 2.17-fold, respectively (Figure 3C). The same treatment was not able to alter the ΔΨm in MDA-MB-231 cells (Figure 3D).

### 3.6. Evaluation of Cell Cycle through the Analysis of DNA Content of Breast Cancer Cells after Treatment with Oxyresveratrol, Doxorubicin, Melphalan, and Associations

**Figure 4:**
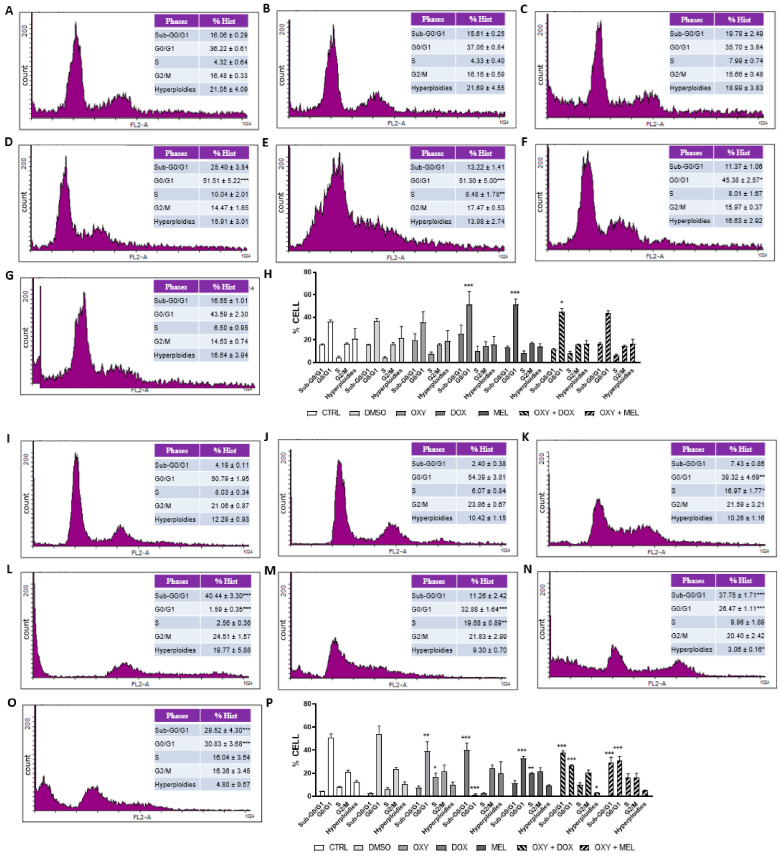
Evaluation of cell cycle through analysis of DNA content of breast cancer cells MCF-7 and MDA-MB-231 after treatment with oxyresveratrol (OXY), doxorubicin (DOX), melphalan (MEL), and associations. The MCF-7 (**A**–**H**) and MDA-MB-231 cells (**I**–**P**) were treated or not with 1% DMSO and IC_50_ of OXY, DOX, MEL, and associations for 24 h, and the cell cycle was evaluated after PI staining by flow cytometry analysis. (**A**,**I**) Control, non-treated cells. (**B**,**J**) Cells treated with 1% DMSO. (**C**,**K**) Cells treated with IC_50_ of OXY. (**D**,**L**) Cells treated with IC_50_ of DOX. (**E**,**M**) Cells treated with IC_50_ of MEL. (**F**,**N**) Cells treated with association of OXY + DOX. (**G**,**O**) Cells treated with association of OXY + MEL for 24 h. Histograms are representative of three independent experiments and show the DNA content (*x* axis, PI fluorescence) versus counts (*y* axis). * *p* < 0.05, ** *p* < 0.001, *** *p* < 0.0001 in relation to control.

Due to the morphological effects of OXY, DOX, MEL, and associations in MCF-7 and MDA-MB-231 cells, we verified whether these compounds would be able to alter the cell cycle. For this, the cells were incubated in the presence or absence of the compounds for 24 h, labeled with cell cycle solution, and analyzed via flow cytometry. The cells treated with DMSO did not alter the cell cycle in relation to the untreated control (Figure 4A,B,I,J,H,P). Our results demonstrate that OXY and OXY + MEL were not able to alter the MCF-7 cell cycle (Figure 4C,G,H). However, DOX, MEL, and the OXY + DOX association were able to significantly alter the G0/G1 phase, with 1.42-, 1.14- and 1.25-fold increases, respectively (Figure 4D–F,H).

In the MDA-MB-231 cells, OXY affected the division pattern of the cells by decreasing the cells in the G0/G1 phase by 1.29-fold and increasing the cells in the S phase by 2.11-fold (Figure 4K,P). The DOX increased the sub-G0/G1 phase by 9.63-fold and decreased the G0/G1 phase by 31.88-fold (Figure 4L,P). MEL decreased the G0/G1 phase by 1.54-fold and increased the S phase by 2.45-fold (Figure 4M,P). The association between OXY + DOX and OXY + MEL increased the sub-G0/G1 phase by 9.0- and 7.03-fold and decreased the G0/G1 phase by 1.91- and 1.64-fold, respectively (Figure 4N–P).

### 3.7. Effects of Oxyresveratrol, Doxorubicin, Melphalan, and Associations on the Type of Cell Death

**Figure 5:**
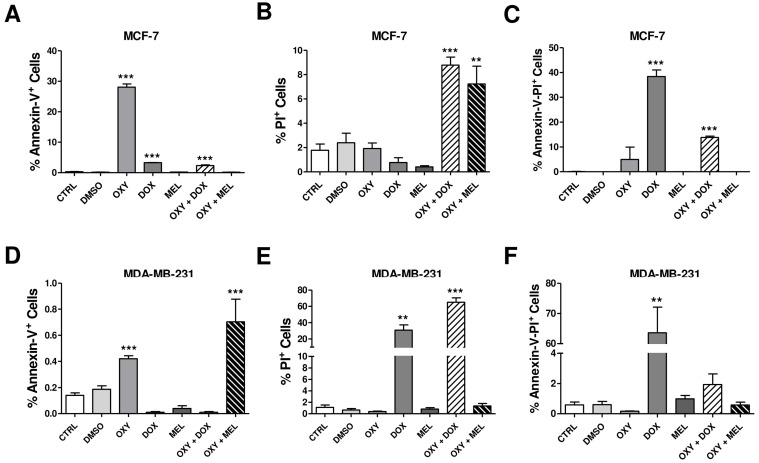
The effects of oxyresveratrol (OXY), doxorubicin (DOX), melphalan (MEL), and associations on type of cell death in MCF-7 and MDA-MB-231 cells. The cells were treated or not with 1% DMSO and IC_50_ of OXY, DOX, MEL, and associations for 24 h. They were then stained with Annexin-V-FITC and propidium iodide (PI), evaluated on a BD FACSCalibur™ flow cytometer (Becton Dickinson), and analyzed using the BD CellQuest™ Pro software. Annexin-V/PI^+^ cells (**A**), PI^+^ cells (**B**) and Annexin-V^+^ cells (**C**) in MCF-7 cells. Annexin-V/PI^+^ cells (**D**), PI^+^ cells (**E**), and Annexin-V^+^ cells (**F**) in MDA-MB-231 cells. The data represent the mean ± SEM of three independent experiments. ** *p* < 0.001, *** *p* < 0.0001 in relation to control.

To discriminate apoptotic and necrotic cell death, the cells were stained with annexin-V-FITC and PI and analyzed via flow cytometry (Figure 5 and Supplementary Figure S1). Our results demonstrate that in MCF-7 cells, OXY, DOX, and OXY + DOX increased the percentage of annexin-V-positive cells by 84.59-, 9.85-, and 7.12-fold in relation to the untreated control (Figure 5A). OXY + DOX and OXY + MEL increased the PI-positive cells by 4.93- and 4.06-fold (Figure 5B). DOX and OXY + DOX increased the annexin-V/PI-positive cells by 369.21- and 133.02-fold, respectively (Figure 5C). In MDA-MB-231 cells, OXY and OXY + MEL increased the percentage of annexin-V-positive cells by 3.00- and 5.02-fold (Figure 5D). DOX and OXY + DOX increased the percentage of PI-positive cells by 28.12- and 59.08-fold (Figure 5E), and DOX increased the percentage of annexin-V/PI-positive cells by 107.80-fold in relation to the untreated control (Figure 5F).

### 3.8. The Effects of Oxyresveratrol, Doxorubicin, Melphalan, and Associations on the Expression of Caspases Involved in Apoptosis

**Figure 6:**
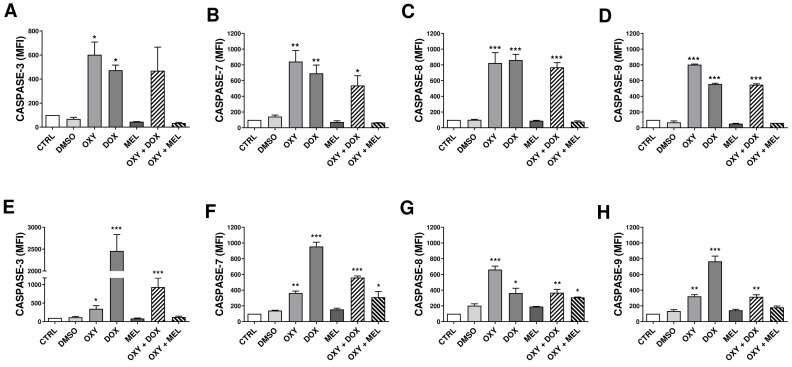
The effects of oxyresveratrol (OXY), doxorubicin (DOX), melphalan (MEL), and associations on immunocytochemistry of caspase-3, caspase-7, caspase-8, and caspase-9 in MCF-7 (**A**–**D**) and MDA-MB-231 cells (**E**–**H**). The cells were treated or not with 1% DMSO and IC_50_ of OXY, DOX, MEL, and associations for 24 h. The cells were fixed, permeabilized, and stained with primary antibody capase-3 (**A**,**E**), caspase-7 (**B**,**F**), caspase-8 (**C**,**G**), and caspase-9 (**D**,**H**), and then stained with fluorescence secondary antibody. Cell fluorescence was evaluated on a BD FACSCalibur™ flow cytometer (Becton Dickinson) and analyzed using the BD CellQuest™ Pro software. The data represent the mean ± SEM of three independent experiments. * *p* < 0.05, ** *p* < 0.001, *** *p* < 0.0001 in relation to control.

To determine the effects of OXY, DOX, MEL, and associations on MCF-7 and MDA-MB-231 cells, caspase-3, caspase-7, caspase-8, and caspase-9 were analyzed via immunocytochemistry (Figure 6). We showed that OXY and DOX increased the levels of caspase-3 in MCF-7 cells by 6.03- and 4.73-fold (Figure 6A). OXY, DOX, and OXY + DOX increased the levels of caspase-7 (Figure 6B) by 8.42-, 6.93-, and 5.38-fold; the levels of caspase-8 (Figure 6C) by 8.24-, 8.61-, and 7.69-fold; and the levels of caspase-9 by 8.03-, 5.56-, and 5.47-fold in MCF-7 cells (Figure 6D). In MDA-MB-231 cells, OXY, DOX, and OXY + DOX increased the levels of caspase-3 by 3.47-, 24.59-, and 9.33-fold (Figure 6E). OXY, DOX, OXY + DOX, and OXY + MEL increased the levels of caspase-7 by 3.64-, 9.56-, 5.60-, and 3.10-fold (Figure 6F). OXY, DOX, OXY + DOX, and OXY + MEL increased the levels of caspase-8 by 6.62-, 3.63-, 3.68-, and 3.10-fold (Figure 6G), and OXY, DOX, OXY + DOX and OXY + MEL increased the levels of caspase-9 by 3.22-, 7.67-, 3.15-, and 1.83-fold compared with the untreated control, respectively (Figure 6H).

### 3.9. Targeted Metabolomics of Oxyresveratrol, Doxorubicin, and Melphalan in the MCF-7 and MDA-MB-231 Cell Lines

**Figure 7:**
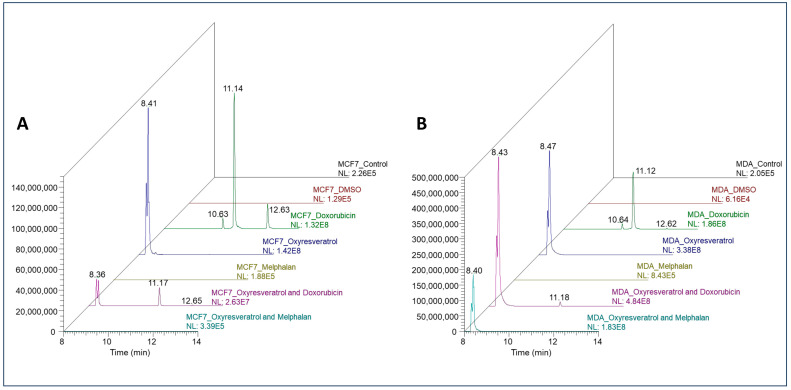
Chromatograms of oxyresveratrol, doxorubicin, melphalan, and its associations, and chromatograms of the putatively identified metabolites (7-deoxydoxorubicinone and doxorubicinol) of doxorubicin in MCF-7 (**A**) and MDA-MB-231 cells (**B**) treated with this substance and its associations.

Oxyresveratrol was observed (Figure 7A,B) in both breast cancer cells and when used combined with the chemotherapeutics (OXY + DOX or OXY + MEL), except for MCF-7 cells treated with OXY + MEL. Doxorubicin was found (Figure 7A,B) in both cell lines treated with this drug and its associations (OXY + DOX).

Additionally, 7-deoxydoxorubicinone (retention time = 12.63 min) and doxorubicinol (retention time = 10.63 min) were putatively identified (Supplementary Table S2) in both cell samples when treated with DOX, and 7-deoxydoxorubicinone was also detected when MCF-7 cells were treated with OXY + DOX.

Melphalan’s standard chromatogram and its full scan mass spectrum show that the method used for the detection of this compound was effective (Supplementary Figure S2), but when the samples were analyzed, melphalan was not detected, thus not showing an incorporation in any of the cell lines treated with this substance, probably due to its low concentration. Chromatograms of OXY and DOX standards and their mass spectra are also available in Supplementary Figure S3 and S4, respectively.

The treatment for both breast cancer cell lines was effective in terms of metabolizing DOX alone or in combination with OXY, and two putative metabolites (7-deoxidoxorubicinone and doxorubicinol) were identified. In contrast, when the cells were incubated with MEL or OXY + MEL and the extracts were analyzed via HPLC/MS, no peak appeared in either cell extract, probably due to the concentration of the compounds used.

It is noteworthy that none of the substances or their possible metabolites under analysis were observed in the control samples, the control with DMSO, or the blanks (Figure 7A,B).

### 3.10. In Silico Analyses of the Oxyresveratrol, Doxorubicin, Melphalan, Doxorubicinol, and 7-Deoxidoxorubicinone

The computational ADMET analysis revealed that OXY has high water solubility and 100% plasma protein binding, as well as high in vitro Mandin Darby Canine Kidney Cell Permeability. It also presents a medium risk of hERG inhibition (Supplementary Table S3). DOX, doxorubicinol, and 7-deoxidoxorubicinone present low Mandin Darby Canine Kidney Cell Permeability and ambiguous hERG inhibition (Supplementary Table S3). OXY, MEL, and 7-deoxidoxorubicinone obey Lipinski’s rule of five. In contrast, DOX and doxorubicinol violate this rule (Supplementary Table S3).

## 4. Discussion

Stilbenes are natural products isolated from several plant species, and resveratrol is by far the most studied among them, with several of its biological effects having already been described, including an anti-cancer effect [3]. Here, we describe the anti-breast cancer effect of OXY, a *trans*-resveratrol derivative. It has been demonstrated that OXY at 10 µM has estrogenic activity and can induce proliferation of MCF-7 cells and upregulation of ERα/β expression. However, it has been shown that OXY, isolated from *S. china* L., presents IC_50_ values of 4.5 and 5.6 µg/mL for MCF-7 and MDA-MB-231 cells, respectively [20,21]. Actually, breast cancer is the type of cancer that most kills women in the world, and in 2020, there were 2.26 million cases [1]. Thus, we sought to better characterize the effect of polyphenols on breast cancer cells by evaluating its anticancer effect of association with two classical chemotherapeutic agents, DOX and MEL, with bioactive compounds for the first time.

It has been commonly assumed that combination chemotherapy for breast cancer, with true synergistic potential, will result in improved response rate, better symptom palliation, and improved survival compared to single-agent chemotherapy [18]. It has been demonstrated that resveratrol improves the anticancer effects of DOX in vitro and in vivo. Furthermore, the combinatorial treatment of 216 nM DOX and 84.6 µM RESV was able to reduce the dose of DOX by 2.5-fold compared to the IC_50_ of DOX alone in MCF-7 and MDA-MB-231 breast cancer cells [7]. Our results demonstrated that 93.61 µM OXY + 5.65 µM DOX in MCF-7 cells and 211.09 µM OXY + 2.034 µM DOX in MDA-MB-231 cells were able to decrease the concentrations of the chemotherapeutic agents DOX and MEL by 4.0- and 16.0-fold, respectively. Meanwhile, the associations of 10.25 µM OXY + 29.25 µM MEL in MCF-7 and 132.93 µM OXY + 15.01 µM MEL in MDA-MB-231 cells were able to decrease the concentrations of the drugs by 5.0- and 16.0-fold, respectively, without them losing their activity when compared separately. Because these associations were synergistic and contained the lowest concentration of the chemotherapeutic agents, they were chosen for the other assays.

We showed that OXY induced morphological changes in MDA-MB-231 cells. In HCT116 and HT-29 colon cancer cells, OXY inhibited cell migration via Snail/E-cadherin expression [22]. In addition, we investigated the cytotoxic effects of OXY, DOX, MEL, and associations over a prolonged period and found, that for 18 days after treatment with IC_50_, they were inhibited. The clonogenic assay allowed us to evaluate the ability of cells to form colonies. This assay detects, after the cytotoxic effect of treatment, whether cells can form and maintain the ability to generate daughter cells. RESV, another stilbene, also has the ability to inhibit colony formation in MDA-MB-231 cells at a concentration of 100 µM [23]. The results of our clonogenic assay support our findings from the MTT assay.

The mechanism of action of DOX is to induce DNA damage, mainly through inhibition of DNA topoisomerase II enzyme, after having induced double-strand DNA breaks [24]. Doxorubicin-induced ROS production could promote the loss of mitochondrial membrane potential, mitochondrial swelling, and outer membrane rupture, which initiates the release of cytochrome c, whose eventual outcome is apoptotic cell death [25]. Corroborating these data, we found that DOX increased the ROS production in MCF-7 and MDA-MB-231 cells. However, the association of OXY + DOX in both cells had the opposite effect. In addition, MEL increased ROS production in MCF-7 cells, whereas in MDA-MB-231, MEL did not alter ROS levels. Previously, our research group has demonstrated that MEL does not alter ROS levels in MDA-MB-231 cells [12].

OXY has an antioxidant effect on many cell types. It has been shown that 30.5 µM of OXY can reduce ROS levels by 3.6-fold in peritoneal macrophages of mice [13]. OXY has also shown protective effects against reactive oxygen and nitrogen species in a murine microglial cell line by considerably decreasing nitric oxide and ROS levels [26]. Consistent with these findings, we also observed that OXY reduced ROS levels in MCF-7 and MDA-MB-231 cells.

In breast cancer, tumor suppressor genes are associated with abnormal histone modification like acetylation and methylation of histone along with DNA. Among these changes, the loss of cell cycle checkpoints may compromise the fidelity of DNA replication prior to cell division, playing an important role during carcinogenesis [27]. Studies have reported that phytochemicals can regulate gene expression by targeting different components of the epigenetic machinery. These epigenetic changes can occur through cell cycle arrest, initiating apoptosis and reactivation of tumor-suppressing genes [28]. Our results demonstrate that OXY was able to alter the G0/G1 and S phase in MDA-MB-231 cells. Furthermore, the association between OXY + DOX and OXY + MEL increased the sub-G0/G1 phase and decreased the G0/G1 phase. It has been reported that MEL’s concentration of 50 µM did not alter the sub-G0/G1 or S phases of the cell cycle in MCF-7 cells; however, its association with RESV was able to significantly increase the sub-G0/G1 and S phases [8]. Moreover, MDA-MB-231 cell treatment with 187.90 µM of MEL for 24 h reduced the G0/G1 phase population and increased the S phase population [12].

In the early stages of apoptosis, cell morphology changes, with size reduction, well-compacted organelles in the cytoplasm, and chromatin condensation [29]. Our light microscopy data show that, after treatment with OXY, the cells shrunk, which is indicative of apoptotic death. In addition, the MDA-MB-231 cells treated with OXY and the MCF-7 and MDA-MB-231 cells treated with OXY + MEL increased the percentage of annexin-V positive cells, which characterizes apoptotic death. Data in the literature have demonstrated the apoptotic effect of OXY on cancer cells. OXY induces autophagy along with apoptosis in neuroblastoma cells, down-regulates the anti-apoptotic Bcl-2, and up-regulates the pro-apoptotic Bax protein [30]. It has been reported that 40 µM OXY for 48 h is able to externalize phosphatidylserine in MDA-MB-231 breast cancer cells, indicating apoptosis [31]. Our immunocytochemistry results also demonstrated that OXY increased the levels of apoptosis activator caspases 8 and 9 and apoptosis executioner caspases 3 and 7 in both MDA-MB-231 and MCF-7 cells.

DOX is an anthracycline frequently used for the treatment of breast cancer. Nonetheless, DOX is also associated with significant cardiac toxicity, which limits the drug’s long-term use. Acute cardiac toxicity after DOX administration manifests as arrhythmias and occurs in up to 26% of the patients, and late cardiac toxicity, potentially lethal, occurs in approximately 1.7% of the patients. Additionally, irreversible DOX-induced cardiomyopathy can occur up to 20 years after the end of treatment [32,33]. Evidence shows that heart diseases resulting from the use of DOX are caused by the accumulation of this chemotherapeutic drug in cardiac mitochondria, generating ROS and promoting apoptosis. Therefore, new protective strategies against cardiotoxicity caused by DOX have been investigated [32,33,34].

A relationship between intracellular levels of DOX and its cytotoxicity has been demonstrated, but there are few reports which have measured the intracellular levels of its main metabolite, doxorubicinol, which has high cardiotoxicity [35,36]. Here, we analyzed the metabolites of OXY, DOX, and MEL present in MCF-7 and MDA-MB-231 cells after treatment. However, we identified only two metabolites of DOX, 7-deoxidoxorubicinone and doxorubicinol. Incredibly, these two metabolites were not identified in cells treated with the association of OXY + DOX. This synergistic association has the possibility of reducing the cardiotoxic effects of DOX. A study on women with breast cancer using vitamin D during chemotherapy with DOX demonstrated a significant reduction in the levels of lactate dehydrogenase, cardiac troponin T, and IL-6, providing evidence of cardioprotective effects of vitamin D by attenuating the deleterious effects caused by DOX treatment [37]. Thus, a strategy based on the inhibition of metabolite formation, such as the conversion of DOX to doxorubicinol, would be considered an approach to reduce cardiotoxicity [38].

To reduce the costs of biological tests and identify possible toxicity, the theoretical approach of in silico pharmacokinetics is largely used in the initial study of new substances [39]. The in silico predictions of OXY, MEL, DOX, and their metabolites were performed via ADMET analysis (Absorption, Distribution, Metabolism, Excretion and Toxicity). CYP2D6, 3A4, 2C9, 2C19, and 2D6 are main cytochrome P450 enzymes that play significant roles in drug metabolism in the liver [40]. The analysis identified OXY, MEL, and 7-deoxydoxorubicinone as CYP3A4 weak substrates and DOX and doxorubicinol as CYP2D6 and CYP3A4 weak substrates. Hyrsova et al. (2019) [41] demonstrated that OXY did not significantly induce CYP3A4 or CYP2B6 expression in primary human hepatocytes. Furthermore, OXY showed inhibitory activity against the enzymes 2C9, 2C19, and 3A4. Chow et al. (2010) [42] demonstrated that RESV acts by inhibiting the phenotypic activity of the same CYPs, which is important for understanding the modulation and metabolism of the bioactive, in addition to identifying possible drug interactions. Breuer et al. (2005) [43] showed that OXY can have protective effects in the brain due to its characteristic of crossing the blood–brain barrier (BBB). Our results demonstrated that OXY has a high rate of penetration of the BBB when compared to other substances. In addition, all drugs were identified as non-Pgp (P-glycoprotein) inhibitors and non-carcinogenic in mice.

## 5. Conclusions

**Figure 8:**
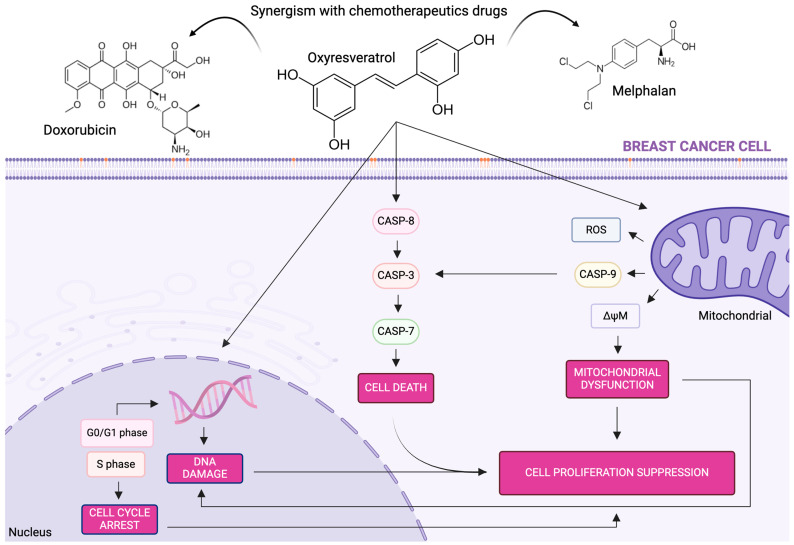
Mechanism of action of oxyresveratrol and its associations with doxorubicin and melphalan in breast cancer cells.

Phytochemicals from food sources have proven to be a good alternative in the treatment of cancer, and are mainly associated with chemotherapy drugs. In this study, we demonstrated the effects of cytotoxicity and synergism in the treatment of MCF-7 and MDA-MB-231 breast cancer cells with OXY and associations of DOX or MEL. The elucidated mechanism of action of OXY and associations in breast cancer cells is summarized in the illustration given in Figure 8. We found that OXY and the synergistic association of OXY + DOX and OXY + MEL led to mitochondrial dysfunction through the alteration of the mitochondrial membrane potential and the production of reactive oxygen species. The treatment also inhibited colony formation; induced DNA damage with cell cycle arrest and necrotic or apoptotic cell death; and upregulated caspases-3, -7, -8, and -9. Thus, this is the first study on the association of the polyphenol OXY and conventional chemotherapeutic agents such as DOX and MEL in breast cancer cells, offering a new strategy for future animal studies.

## Figures and Tables

**Table 1:** Cytotoxicity results for oxyresveratrol, doxorubicin, and melphalan in non-tumor breast MCF-10A cells and breast cancer MCF-7 and MDA-MB-231 cells.

DRUGS	IC_50_ MCF-10A (µM)	IC_50_ MCF-7 (µM)	SI *	IC_50_ MDA-MB-231 (µM)	SI ^#^
OXYRESVERATROL	>300	164.10 ± 19.17	>1.83	287.08 ± 11.09	>1.05
DOXORUBICIN	26.54 ± 4.81	22.60 ± 1.61	1.17	32.55 ± 17.80	0.82
MELPHALAN	195.97 ± 11.07	155.70 ± 6.21	1.26	240.26 ± 25.46	0.82

IC_50_—inhibitory concentration to 50% of the cells. * The selectivity index (SI) was calculated as the ratio of IC_50_ of MCF-10A cells to IC_50_ of MCF-7 cells. ^#^ The selectivity index (SI) was calculated as the ratio of IC_50_ of MCF-10A cells to IC_50_ of MDA-MB-231 cells.

**Table 2:** Combination index of oxyresveratrol with doxorubicin (OXY + DOX) and oxyresveratrol with melphalan (OXY + MEL) in MCF-7 and MDA-MB-231 breast cancer cells.

CELL LINES	COMBINATION DOSES (µM)	* FIC	EFFECTS
**MCF-7**	OXY 93.61 + DOX 5.65	0.82	Moderate Synergism
	OXY 42.95 + DOX 11.30	0.76	Moderate Synergism
	OXY 10.25 + MEL 29.25	0.25	Strong Synergism
	OXY 20.51 + MEL 31.34	0.32	Synergism
	OXY 41.02 + MEL 33.40	0.46	Synergism
	OXY 82.05 + MEL 32.30	0.63	Synergism
**MDA-MB-231**	OXY 211.09 + DOX 2.03	0.80	Moderate Synergism
	OXY 208.09 + DOX 8.14	0.97	-
	OXY 202.30 + DOX 16.27	1.20	-
	OXY 132.93 + MEL 15.01	0.52	Synergism
	OXY 116.30 + MEL 30.03	0.53	Synergism
	OXY 87.69 + MEL 60.06	0.55	Synergism
	OXY 37.69 + MEL 120.13	0.63	Synergism

* FIC—fractional inhibitory concentration.

## Data Availability

Data is contained within the article and Supplementary Materials.

## Supplementary Materials

The following supporting information can be downloaded at: https://www.mdpi.com/article/10.3390/pharmaceutics16070873/s1, Figure S1: The effects of oxyresveratrol (OXY), doxorubicin (DOX), melphalan (MEL), and associations on the type of cell death in MCF-7 and MDA-MB-231 cells; Figure S2: Melphalan’s analytical standard chromatogram and its protonated precursor ion mass spectrum; Figure S3: Oxyresveratrol’s analytical standard chromatogram and its protonated precursor ion mass spectrum; Figure S4: Doxorubicin’s analytical standard chromatogram and its protonated precursor ion mass spectrum; Table S1: Oyresveratrol, doxorubicin, and melphalan standards and their metabolites investigated on the present study. Their molecular formula (MF), monoisotopic masses, and theoretical *m/z* values in a positive electrospray ionization mode are listed. Table S2: Retention time of oxyresveratrol, melphalan, doxorubicinol, doxorubicin, and 7-deoxydoxorubicinone; Table S3: In silico ADMET data of oxyresveratrol, doxorubicin, melphalan, doxorubicinol, and 7-Deoxydoxorubicinone.
