# Supplementary material for: Oxyresveratrol in Breast Cancer Cells: Synergistic Effect with Chemotherapeutics Doxorubicin or Melphalan on Proliferation, Cell Cycle Arrest, and Cell Death
Source: Pharmaceutics. 2024 Jun 29;16(7):873. doi: 10.3390/pharmaceutics16070873 (PMC11279446; doi:10.3390/pharmaceutics16070873)
Supplement: Supplementary file 1 [file pharmaceutics-16-00873-s001.zip › pharmaceutics-3047422-supplementary.pdf]

Oxyresveratrol in breast cancer cells: synergistic effect with chemotherapeutics doxorubicin or melphalan on proliferation, cell cycle arrest and cell death

Carlos Luan Alves Passos[a], Christian Ferreira[a], Aline Gabrielle Alves de Carvalho[b], Jerson Lima Silva[c], Rafael Garrett[b], Eliane Fialho[a*]

[a] Institute of Nutrition Josué de Castro, Federal University of Rio de Janeiro, Rio de Janeiro, RJ 21941-902, Brazil.

[b] Institute of Chemistry, Federal University of Rio de Janeiro, Rio de Janeiro, RJ 21941-909, Brazil.

[c] Institute of Medical Biochemistry Leopoldo De Meis, Federal University of Rio de Janeiro, Rio de Janeiro, RJ 21941-902, Brazil.

*Corresponding author:

Prof[a]. Eliane Fialho, PhD. Departamento de Nutrição Básica e Experimental, Instituto de Nutrição Josué de Castro, Centro de Ciências da Saúde, Universidade Federal do Rio de Janeiro, UFRJ, Caixa Postal 68041, Cidade Universitária, Ilha do Fundão, Rio de Janeiro, CEP 21941-902, Brazil. E-mail adress: fialho@nutricao.ufrj.br. Fax number: + 55 21 2280 8343. Tel number: +55 21 3938 6799.

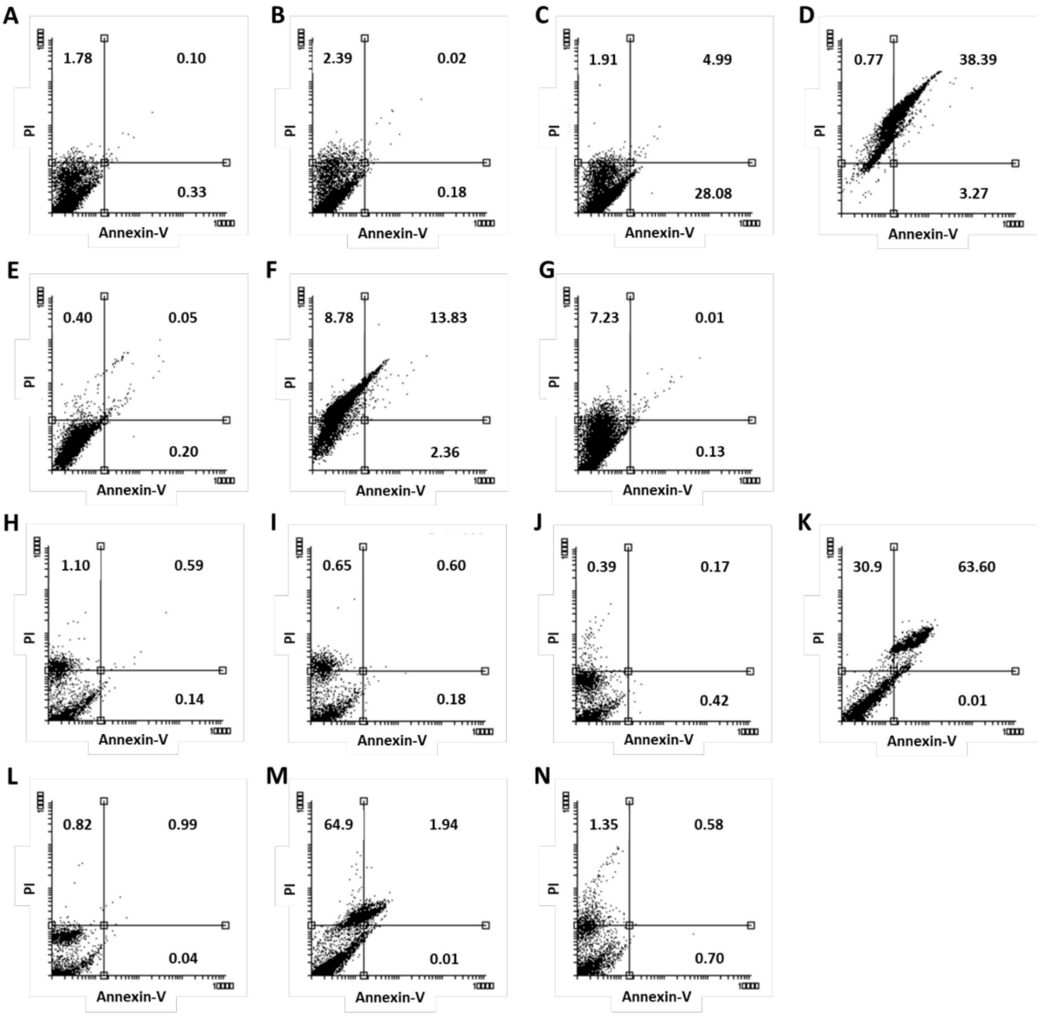

Fig. S1 - Effect of Oxyresveratrol (OXY), Doxorubicin (DOX), Melphalan (MEL) and associations on type of cell death in MCF-7 and MDA-MB-231 cells. (**A and H**) Control, not treated cells. (**B and I**) Cells treated with 1% DMSO. (**C and J**) Cells treated with IC_{50} of OXY. (**D and K**) Cells treated with IC_{50} of DOX. (**E and L**) Cells treated with IC_{50} of MEL. (**F and M**) Cells treated with Association of OXY + DOX. (**G and N**) Cells treated with Association of OXY + MEL for 24 hours. They were then stained with Annexin-V-FITC and propidium iodide (PI), evaluated on a BD FACSCalibur™ flow cytometer (Becton Dickinson) and analyzed using BD CellQuest™ Pro software. The dot plot flow cytometry data represent the mean ± SEM of three independent experiments.

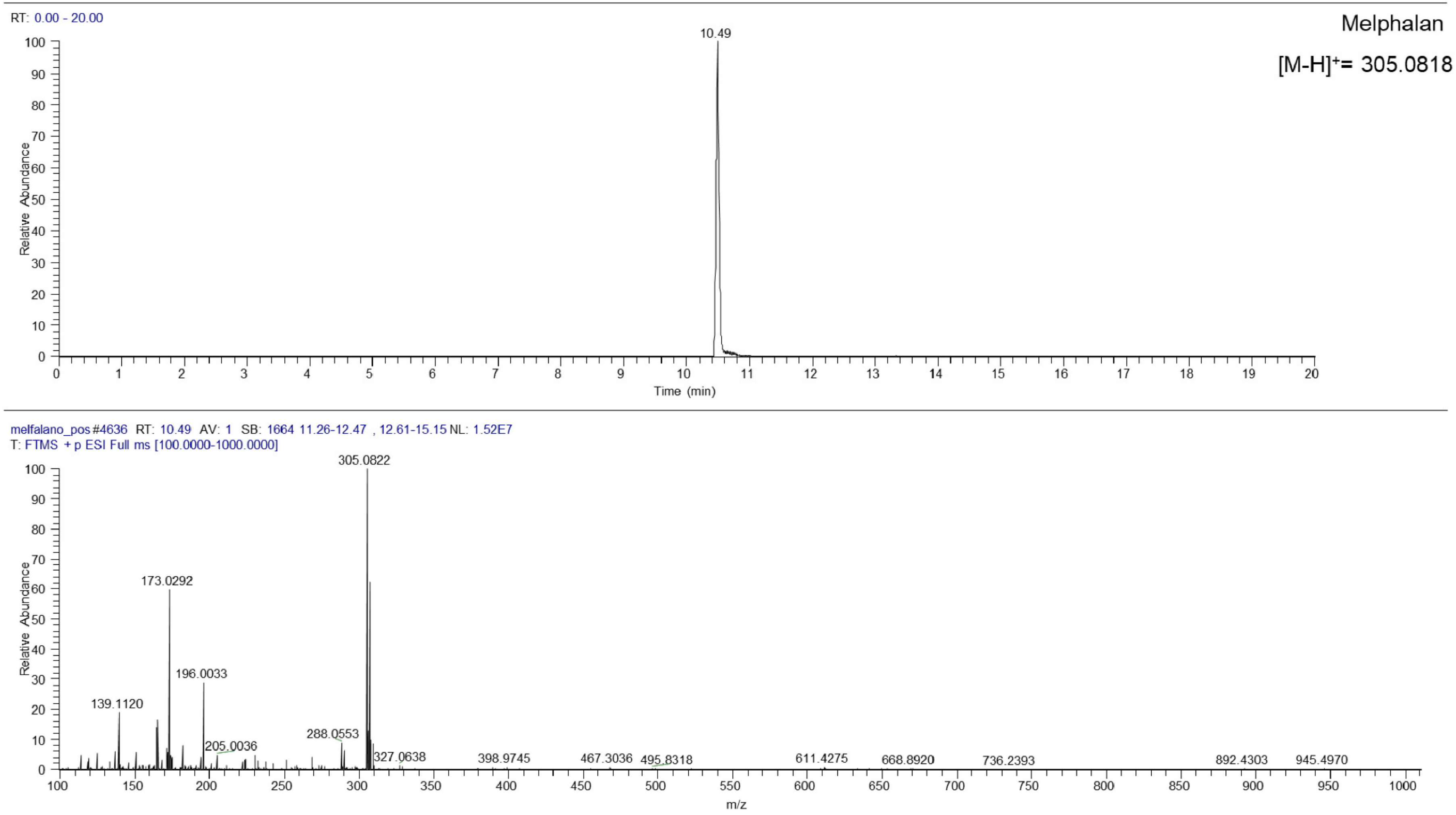

Fig. S2 - Melphalan's analytical standard chromatogram and its protonated precursor ion mass spectrum.

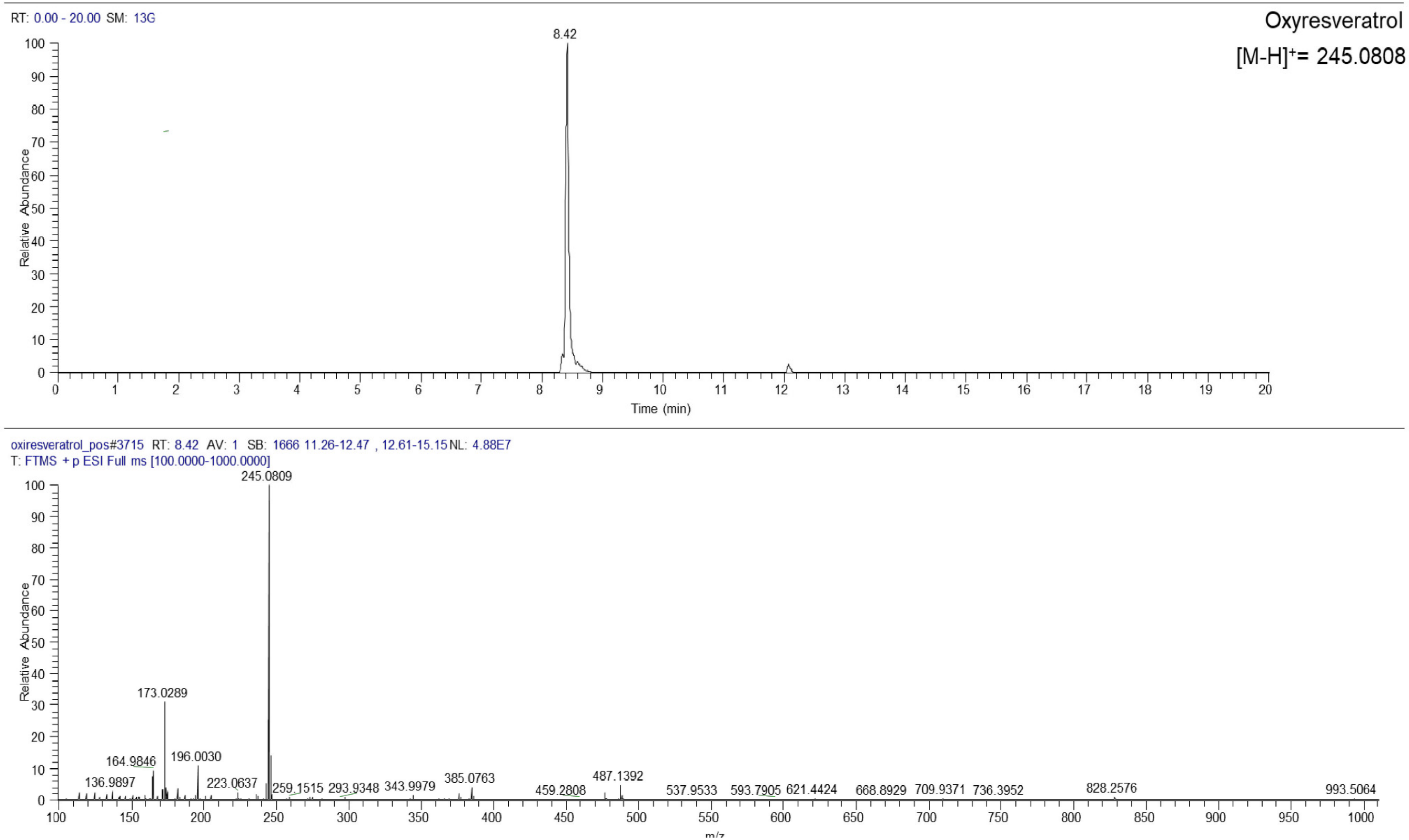

Fig. S3 - Oxyresveratrol's analytical standard chromatogram and its protonated precursor ion mass spectrum.

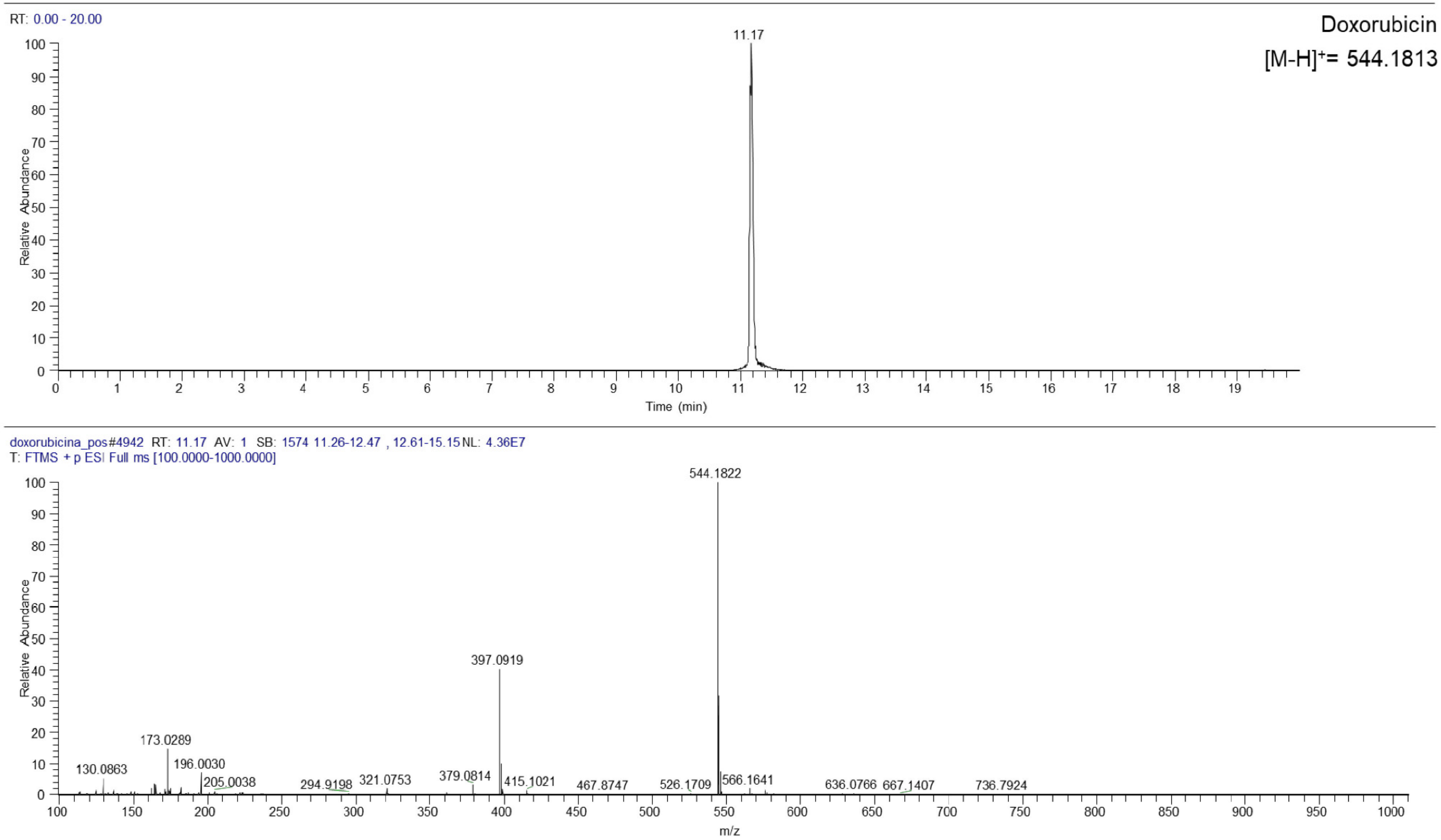

Fig. S4 - Doxorubicin’s analytical standard chromatogram and its protonated precursor ion mass spectrum.

Table S1. Oyresveratrol, Doxorubicin and Melphalan standards and their metabolites investigated on the present study. Their molecular formula (MF), monoisotopic masses and theoretical *m/z* values in positive electrospray ionization mode are listed.

Analyte standard	molecular formula	monoisotopic masses	$[M+H]^+$
**Oxyresveratrol**	$C_{14}H_{12}O_4$	244.0736	245.0808
**Melphalan**	$C_{13}H_{18}Cl_2N_2O_2$	304.0745	305.0818
**Doxorubicin**	$C_{27}H_{29}NO_{11}$	543.1741	544.1813
**Metabolyte**	**molecular formula**	**monoisotopic masses**	$[M+H]^+$
**Resveratrol**	$C_{14}H_{12}O_3$	228.0786	229.0859
**Oxyresveratrol-glucuronide**	$C_{20}H_{20}O_{10}$	420.1056	421.1129
**dihydro-oxyresveratrol monoglucuronide**	$C_{20}H_{22}O_{10}$	422.1213	423.1285
**Oxyresveratrol monosulfate**	$C_{14}H_{13}SO_7$	325.0382	326.0454
**Dihydro-oxyresveratrol monosulfate**	$C_{14}H_{15}SO_7$	327.0532	328.0611
**Oxidation derivative of oxyresveratrol monoglucuronide**	$C_{20}H_{18}O_{10}$	418.0899	419.0973
**Methylated oxyresveratrol monoglucuronide**	$C_{21}H_{22}O_{10}$	434.1212	435.1285
**Hydroxylate of dihydro-oxyresveratrol monoglucuronide**	$C_{20}H_{22}O_{10}$	422.1212	423.1285
**2,3,4,5-Tetrahydroxybibenzyl**	$C_{14}H_{14}O_4$	246.0892	247.0965
**Oxyresveratrol-2-O-β-D-glucopyranoside**	$C_{20}H_{22}O_9$	406.1264	407.1337
**Doxorubicinol**	$C_{27}H_{31}NO_{11}$	545.1897	546.1969
**Doxorubicinone**	$C_{21}H_{18}O_9$	414.0951	415.1023
**7-Deoxydoxorubicinone**	$C_{21}H_{18}O_8$	398.1002	399.1074
**Doxorubicinolone**	$C_{21}H_{20}O_9$	416.1107	417.1180
**Monohydroxymelphalan**	$C_{13}H_{19}ClN_2O_3$	286.1084	287.1156
**Dihydroxymelphalan**	$C_{13}H_{20}N_2O_4$	268.1423	269.1495

Table S2 – Retention time of Oxyresveratrol, Melphalan, Doxorubicinol, Doxorubicin and 7-deoxydoxorubicinone.

Retention time (min)	Substance	$[M+H]^+$
8.41	Oxyresveratrol	245.0808
10.49	Melphalan	305.0818
10.63	Doxorubicinol	546.1969
11.17	Doxorubicin	544.18.13
12.63	7-Deoxydoxorubicinone	399.1074

Table S3. *In silico* ADMET data of Oxyresveratrol, Doxorubicin, Melphalan, Doxorubicinol and 7-Deoxydoxorubicinone.

In silico data	Oxyresveratrol	Doxorubicin	Melphalan	Doxorubicinol	7-Deoxydoxorubicinone
**Solubility in water (mg/L)**	726.73	112.69	297.03	232.08	161.74
**Buffer solubility (mg/L)**	28.04	15714.5	1367.95	22325.9	2630.17
**AlogP98**	2.74	-0.23	2.34	-0.26	1.37
**Plasma protein binding (%)**	100.0	32.78	48.00	34.42	76.58
**Human intestinal absorption (%)**	81.96	31.95	97.16	24.93	75.78
**Intestinal cell permeability (nm/s)**	19.84	17.72	20.95	17.71	20.13
**MDCK (nm/s)**	44.35	1.02	68.99	1.10	5.76
**Skin permeability (logKp, cm/h)**	-3.42	-4.69	-2.61	-4.78	-4.29
**Blood-Brain Barrier penetration**	0.92	0.032	0.016	0.031	0.036
**Pgp inhibition**	non	non	non	non	non
**CYP inhibition**	2C9, 2C19, 3A4	2C9, 2C19, 3A4	2D6	2C9, 2C19, 2D6, 3A4	2C9, 2C19, 3A4
**CYP substrate**	3A4 (weakly)	2D6, 3A4 (weakly)	3A4 (weakly)	2D6, 3A4 (weakly)	3A4 (weakly)
**Rule of 5**	suitable	violated	suitable	violated	suitable
**Ames test**	mutagen	non-mutagen	mutagen	mutagen	non-mutagen
**Carcinogenicity in mouse**	negative	negative	negative	negative	negative
**hERG inhibition**	medium risk	ambiguous	low risk	ambiguous	ambiguous

AlogP98 (the logarithm of the partition coefficient between n-octanol and water, MDCK: *In vitro* Mandin Darby Canine Kidney Cell Permeability, Pgp: P- glycoprotein inhibition, CYP: cytochrome P5450 isoforms and hERG: Inhibition of the human Ether-a-go-go Related Gene (potassium channel).
